# Organ-specific accuracy of [^18^F]FDG-PET/CT in identifying immune-related adverse events in patients with high-risk melanoma treated with adjuvant immune checkpoint inhibitor

**DOI:** 10.1007/s11604-024-01554-y

**Published:** 2024-03-20

**Authors:** Birte Molvik Gideonse, Magnus Birkeland, Mie Holm Vilstrup, Peter Grupe, Mohammad Naghavi-Behzad, Christina H. Ruhlmann, Oke Gerke, Malene Grubbe Hildebrandt

**Affiliations:** 1 Department of Clinical Research, University of Southern Denmark, Odense, Denmark; 2 Department of Nuclear Medicine, Odense University Hospital, Odense, Denmark; 3 Department of Radiology and Nuclear Medicine, Esbjerg Hospital, Esbjerg, Denmark; 4 Centre for Personalized Response Monitoring in Oncology, Odense University Hospital, Odense, Denmark; 5 Department of Oncology, Odense University Hospital, Odense, Denmark; 6 Centre for Innovative Medical Technology, Odense University Hospital, Odense, Denmark

**Keywords:** [^18^F]FDG-PET/CT, Immune-related adverse events, irAEs, Melanoma, Immune checkpoint inhibitor

## Abstract

**Purpose:**

This study aimed to determine the organ-specific accuracy of [^18^F]FDG-PET/CT in identifying immune-related adverse events (irAEs) in patients with high-risk (stage III/IV) surgically resected melanoma treated with an adjuvant immune checkpoint inhibitor (ICI) and determine the incidence of irAEs within the first year after starting treatment.

**Materials and methods:**

This registry-based study included individuals who had undergone surgical removal of melanoma and were undergoing adjuvant ICI treatment (either nivolumab or pembrolizumab). The study specifically enrolled patients who had undergone both a baseline and at least one subsequent follow-up [^18^F]FDG-PET/CT scan. Follow-up scans were performed every third month in the first year after surgery to screen for disease recurrence. We retrospectively compared the follow-up scans with baseline scans to identify irAEs. Clinical information on irAEs was obtained from medical records and served as a reference standard for determining the accuracy of [^18^F]FDG-PET/CT.

**Results:**

A total of 123 patients with 363 [^18^F]FDG-PET/CT scans were included, and 65 patients (52.8%) developed irAEs. In decreasing order, the organ-specific incidences of irAEs were: skin 26/65 (40%), muscle and joints 21/65 (32.3%), intestines 13/65 (20%), thyroid gland 12/65 (18.5%), lungs 4/65 (6.2%), and heart 2/65 (3.1%). The sensitivities and specificities of [^18^F]FDG-PET/CT for diagnosing irAEs were: skin 19% (95% CI: 7–39%) and 95% (88–98%), muscles and joints 71% (48–89%) and 83% (75–90%), intestines 100% (75–100%) and 85% (77–91%); thyroid gland 92% (62–99%) and 95% (89–98%), lungs 75% (19–99%) and 90% (83–95%), and heart 50% (13–99%) and 97% (92–99%), respectively.

**Conclusion:**

[^18^F]FDG-PET/CT generally had moderate to high sensitivities (except for skin and heart) and specificities in diagnosing irAEs in patients receiving adjuvant ICI; this could be suggested to be systematically assessed and reported in scan reports.

## Introduction

Melanoma is a type of skin cancer with a high mortality rate due to its high metastatic potential and aggressive nature. Despite its increasing incidence, the survival rate in the more advanced stages remains moderate, with a 5-year survival rate of approximately 50% in patients with distant metastases [1, 2]. One of the most efficient therapeutic strategies for patients with high-risk melanoma is treatment with adjuvant immune checkpoint inhibitors (ICIs) because of the immunogenicity and increased recurrence-free survival [1–4]. Although patients undergoing adjuvant treatment with ICIs have a significantly better prognosis [5], approximately 10% discontinue treatment due to therapy-related adverse events [6].

After introducing adjuvant programmed cell death protein 1 (PD-1) as a first-line treatment for patients with resected high-risk melanoma, the overall survival has increased significantly [4, 7]. Despite an improved prognosis, patients who receive adjuvant treatment with PD-1 inhibitors (nivolumab or pembrolizumab) are likely to experience immune-related adverse events (irAEs) [5]. Approximately 14% of patients receiving adjuvant ICIs experience severe treatment-related adverse events (grade 3–5) [3, 6, 8]. IrAEs commonly arise within a few weeks or months after treatment initiation, although they can occur at any point during or after administering ICI [6]. Early detection and identification of irAEs and prompt treatment initiation can improve clinical outcomes and lower the risk of sequelae [9, 10]. Up to 50% of patients develop dermatological adverse events, the most prevalent irAE in patients receiving immunotherapy. Other common sites of inflammation include the gastrointestinal tract (40%), thyroid gland (6–20%), and joints (3.5%); inflammation at these sites can lead to colitis, thyroiditis, and arthritis, respectively [11–14].

Since [^18^F]Fluoro-d-glucose-positron emission tomography/computed tomography ([^18^F]FDG-PET/CT) can distinguish between healthy and pathological tissues by detecting the cell’s metabolic glucose activity [15], it can be used to detect recurrence of malignant disease, including melanoma [16, 17], with a sensitivity of 87–97% for diagnosing recurrent disease [18, 19]. By the ability to detect reactivation of the immune system, [^18^F]FDG-PET/CT can potentially be used to diagnose irAEs caused by immunotherapy. This may result in earlier detection of irAEs, thereby potentially improving clinical management and quality of life. [^18^F]FDG-PET/CT is absent in international guidelines as a modality of choice to screen for recurrence in patients with melanoma, even though it has been recommended in local guidelines, including the Danish guidelines, to monitor patients with high-risk melanoma [20]. However, to the best of our knowledge, the diagnostic accuracy of [^18^F]FDG-PET/CT in identifying irAEs in patients with melanoma has not yet been established.

The aim of this study was to determine the accuracy of [^18^F]FDG-PET/CT in identifying irAEs in patients with high-risk melanoma receiving an ICI and to determine the incidence of irAEs associated. Specifically, our objectives were to diagnose organ-specific irAEs using repetitive [^18^F]FDG-PET/CT within the first year of treatment and compare the results with the irAEs reported in the patient’s medical records. We hypothesise that [^18^F]FDG-PET/CT could be utilised as a modality to diagnose irAEs in patients with high-risk melanoma undergoing adjuvant ICIs.

## Materials and methods

In this register-based study on diagnostic accuracy of organ-specific irAEs, we re-analysed [^18^F]FDG-PET/CT data for which the incidence of recurrence has previously been published [18].

### Ethics

This was a non-interventional study on diagnostic accuracy; therefore, the researchers did not influence the patients. Written informed consent was obtained from all patients prior to data collection and transmission to the Danish Metastatic Melanoma Database (DAMMED) [21]. The project was reported to the General Data Protection Regulation, Region of Southern Denmark (GDPR; Journal number 20/59961). The ethical standards of the Institutional and National Research Committee were obeyed, in addition to conformance with the Helsinki Declaration and its subsequent amendments.

### Patients

Patients who had undergone radical resection for high-risk (stage III or IV) melanoma and were treated with an adjuvant ICI were considered eligible for inclusion. All patients had been admitted to the Department of Oncology at Odense University Hospital (OUH), Denmark, between November 2018 and February 2021. Treatment with an ICI was administered every third or fourth week for up to 1 year or until reports of unacceptable adverse events, disease recurrence, or patients’ wish for withdrawal. Follow-up scans were performed approximately every third month in the first year after surgery to detect recurrence, in accordance with the Danish guidelines for patients with resected high-risk melanoma [20]. The patients were required to have received at least one dose of adjuvant immunotherapy (nivolumab or pembrolizumab), should have undergone a baseline [^18^F]FDG-PET/CT scan, and had at least one follow-up scan at one or a combination of the Departments of Nuclear Medicine at Odense, Esbjerg, and Vejle Hospitals (Denmark) to meet the inclusion criteria of this study. Patients with melanoma stage I or II, as well as those with other malignancies and/or the absence of an [^18^F]FDG-PET/CT follow-up or baseline scan, were not included in this study.

### Data extraction

Information regarding the included patients was extracted from the DAMMED and managed using Research Electronic Data Capture (REDCap) [22, 23]. All necessary information regarding the patient's demographic and clinical status was collected from the patients' medical records and registered in REDCap. Detailed information on the immunotherapy treatment lines and doses were also collected. Moreover, we reported the specific dose at which adverse events first emerged, whether or not the patient needed treatment, as well as a continuous/discontinuous treatment regimen after the onset of irAEs. [^18^F]FDG-PET/CT images were obtained from the Departments of Nuclear Medicine at Odense, Esbjerg, and Vejle Hospitals (Denmark) and were visually analysed by three experienced nuclear medicine specialists without grading.

### Immune-related adverse events

Reports of irAEs and their time of onset were extracted from medical records and classified based on the affected organ system. At every clinical appointment irAE were systematically addressed and recorded. To be defined as an irAE, the patient must have received first-line treatment for it or discontinued immunotherapy due to its negative effect. The irAEs were classified dichotomously, present or not. Blood samples were obtained prior to each dose of immunotherapy and repeated on suspicion of irAEs. Some irAEs were discovered by laboratory screening tests (e.g., thyroiditis) or by visual examination, as in the case of dermatological irAEs. Some patients experienced clinical symptoms that could lead to the diagnosis of the irAE, such as diarrhoea in colitis, myalgia or arthralgia in muscle and joints, and pruritus in dermatological manifestations. In the event of an irAE, the patients were assessed and treated according to guidelines endorsed by the Danish Society for Clinical Oncology [24].

### Scan report interpretation

The interpretation of [^18^F]FDG-PET/CT scans for potential irAEs was meticulously performed by a team of three experienced nuclear medicine specialists (MHV, PG, and MGH) using a standardized protocol. This interpretative process adhered to established guidelines and protocols [25], involving a systematic review of each organ within the [^18^F]FDG-PET/CT scans. To ensure precise differentiation between hypermetabolic abnormalities associated with irAEs and those linked to malignant lesions, the interpreting clinicians possessed an in-depth understanding of melanoma presentation patterns on scans. Additionally, these specialists followed well-recognized principles from the field of nuclear medicine to distinguish ‘hypermetabolic irAEs’ from normal hypermetabolic tissues [26–28]. For example, when evaluating muscle, lymph nodes, and joints for irAEs, the specialists incorporated technical criteria involving the intensity, distribution, and spatial extent of FDG uptake. In cases of muscle and joint irAEs, localized hypermetabolic regions adjacent to affected areas were meticulously assessed, considering both the intensity and patterns of FDG uptake. Similarly, lymph nodes were evaluated based on the extent and intensity of FDG uptake in relation to anatomical context. Notably, the pituitary gland, although not extensively analysed in this study, exhibited potential scan changes linked to pituitary irAEs, possibly indicating immune-related inflammation within this region. It is pertinent to note that the interpreting clinicians maintained a blinded approach to patients’ medical records, while being privy to the referral text and image reports generated during clinic visits. To detect new metabolic glucose activity indicative of irAEs, each follow-up scan was meticulously compared with the corresponding previous scan. The baseline scan, conducted prior to the initiation of immunotherapy, was succeeded by subsequent scans at intervals of approximately three, six, nine, and 12 months after the commencement of immunotherapy. This rigorous scan interpretation process facilitated a precise assessment of the organ-specific accuracy of [^18^F]FDG-PET/CT scans in identifying irAEs [29, 30].

### Outcome measures

Clinical information about irAEs from medical records served as a reference standard for determining the accuracy of [^18^F]FDG-PET/CT scans in diagnosing irAEs. Patients were initially divided into two groups: those who had clinical irAEs and those who did not, and the site for the adverse event was registered. They were subsequently divided into groups based on whether they had increased organ-specific FDG uptake that could reflect irAEs or not. The results were then divided into four categories per organ to determine organ-specific accuracy: true positive, false positive, true negative, and false negative. Accuracy measures of sensitivity, specificity, and positive and negative predictive values (PPV and NPV) were determined for organ-specific irAEs. We defined true positives as scans detecting irAEs in patients clinically diagnosed with irAEs, who subsequently received a clinical intervention such as systemic treatment for it and/or the ICI was ended due to the irAE. False positives were identified as instances where irAEs were detected on [^18^F]FDG-PET/CT scans without a corresponding clinical intervention. True negatives were characterized as scans without detectable irAEs, and these patients did not have any clinical intervention related to irAEsymptoms. False negatives were determined as cases where patients received treatment for irAEs and/or ended the ICI based on clinical symptoms, despite the [^18^F]FDG-PET/CT scan not revealing any signs of irAEs.

### [^18^F]FDG-PET/CT

[^18^F]FDG-PET/CT scans were primarily performed to screen for disease recurrence after resection of high-risk melanoma. The [^18^F]FDG-PET/CT scans were performed at hospitals throughout the Region of Southern Denmark, including Odense, Vejle, and Esbjerg. In addition to [^18^F]FDG-PET scan, either a low-dose CT or diagnostic contrast-enhanced CT were employed. Whole-body PET/CT scans were performed in accordance with guidelines from the European Association of Nuclear Medicine (EANM) [25]. In preparation, the patients were instructed to fast for a minimum of 4 h prior to ^18^F-FDG injection. Approximately 60 min before the scan, the patients were injected with 4 MBq of ^18^F-FDG per kilogram body weight.

For the majority of patients (Odense University Hospital), a GE Discovery 710 PET/CT scanner was used to collect the data. The PET scan was carried out using a standard whole-body acquisition methodology that took 2.5 min per bed position and a scan field of view of 70 cm. PET data were reconstructed using iterative 3D OSEM (3 iterations, 24 subsets) with corrections for time-of-flight (GE VPFX) and point-spread-blurring into transaxial slices with a matrix size of 256 × 256 (pixel size 2.74 mm) and a slice thickness of 3.75 mm (GE sharpIR). A particular extremely low-dose helical CT attenuation correction scan was used for attenuation correction. Following the PET scan with in vivo contrast (ultravist 370 I/ml), a helical diagnostic CT scan was obtained using a typical CT procedure with a scan field of view of 70 cm. Data were reconstructed with a standard filter into transaxial slices with a field of view of 50 cm, matrix size of 512 × 512 (pixel size 0.98 mm), and a slice thickness of 3.75 mm. CT, PET, and fused PET/CT data were analysed using a GE Advantage Workstation v. 4.4. A radiologist interpreted the CT scan, while a nuclear medicine specialist interpreted the PET scan. Details for scan methodology for patients from Vejle and Esbjerg hospitals can be seen in our previous publication [18].

### Interpretation of irAEs in [^18^F]FDG-PET/CT

The interpretation of [^18^F]FDG-PET/CT scans for potential irAEs was conducted in accordance with established criteria. A primary criterion involved visually identifying the presence of increased FDG uptake in specific organs or regions compared to the organ-specific FDG activity at baseline. This visual-based assessment systematically evaluated FDG uptake intensity across all organs, comparing it to the baseline or subsequent scan without quantification analyses. Crucially, the diagnosis of irAEs relied on the appearance of new or higher FDG uptake. For instance, if a patient exhibited high uptake, such as in the thyroid or colon at baseline, only an increase in FDG uptake was considered indicative of an irAE. Moreover, the interpretation process included a meticulous examination of the pattern and distribution of FDG uptake within the organs. Various uptake patterns, including focal, diffuse, or multifocal, were considered in conjunction with the anatomical location of the lesions. Focal intense uptake, particularly when observed adjacent to affected areas such as muscles, joints, or lymph nodes, was deemed indicative of potential irAEs [31].

### Statistical analyses

Descriptive statistics were applied to the dataset based on the nature of the variables: categorical variables were presented as frequencies and percentages, while continuous variables were summarized using medians and ranges (minimum–maximum). To assess the accuracy of [^18^F]FDG-PET/CT in identifying irAEs, organ-specific accuracy measures were determined, accompanied by corresponding 95% confidence intervals (95% CI). The sensitivity, specificity, PPV, and NPV were computed for each organ involved. The statistical software STATA/BE (version 17.0, StataCorp, College Station, USA) was utilized for conducting all statistical analyses.

## Results

**Fig. 1:**
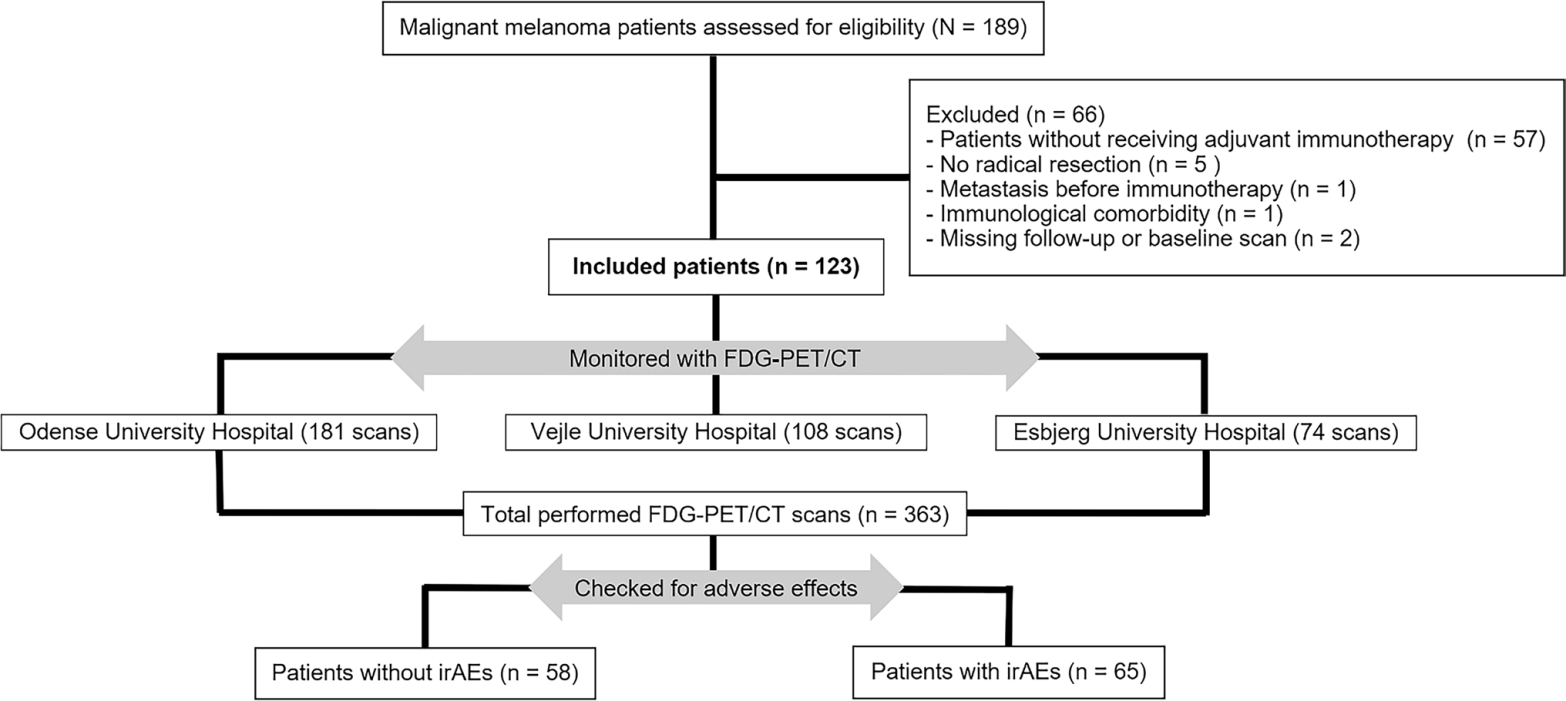
Flowchart presenting eligible, included, and excluded patients of the study (irAEs: Immune-related adverse events)

Of the 189 patients with surgically resected high-risk melanoma treated with adjuvant ICI, 66 were excluded, and a total of 123 patients with 363 [^18^F]FDG-PET/CT scans met the inclusion criteria (Fig. 1). Patients underwent a median of four (range 2–6) [^18^F]FDG-PET/CT scans, giving a median timeline of nine months of follow-up. At baseline, the eligible population ranged from 17 to 83 years of age, with the elderly population accounting for the majority, resulting in a median age of 62 years. Most of the patients (60.2%) were men. One patient switched to pembrolizumab due to an allergic reaction, while the remaining patients received adjuvant treatment with nivolumab. The baseline characteristics of the included patients are described in Table 1.

**Table 1:** Baseline characteristics of 123 included patients with melanoma receiving adjuvant immunotherapy

Characteristics	Results*
Age (year)	62 (17–83)
Sex	
Male	74 (60.2)
Female	49 (39.8)
Stage at surgery	
IIIA	16 (13)
IIIB	38 (30.8)
IIIC	55 (44.7)
IIID	0 (0)
IV	14 (11.4)
Radicality of the surgical resection	
R0	103 (83.7)
R1	19 (15.5)
Unknown	1 (0.8)
Number of treatment series	7 (1–13)
Number of performed scans	4 (2–6)

*Data was shown as median (range) and frequency (%)

### Incidence of adverse events

**Fig. 2:**
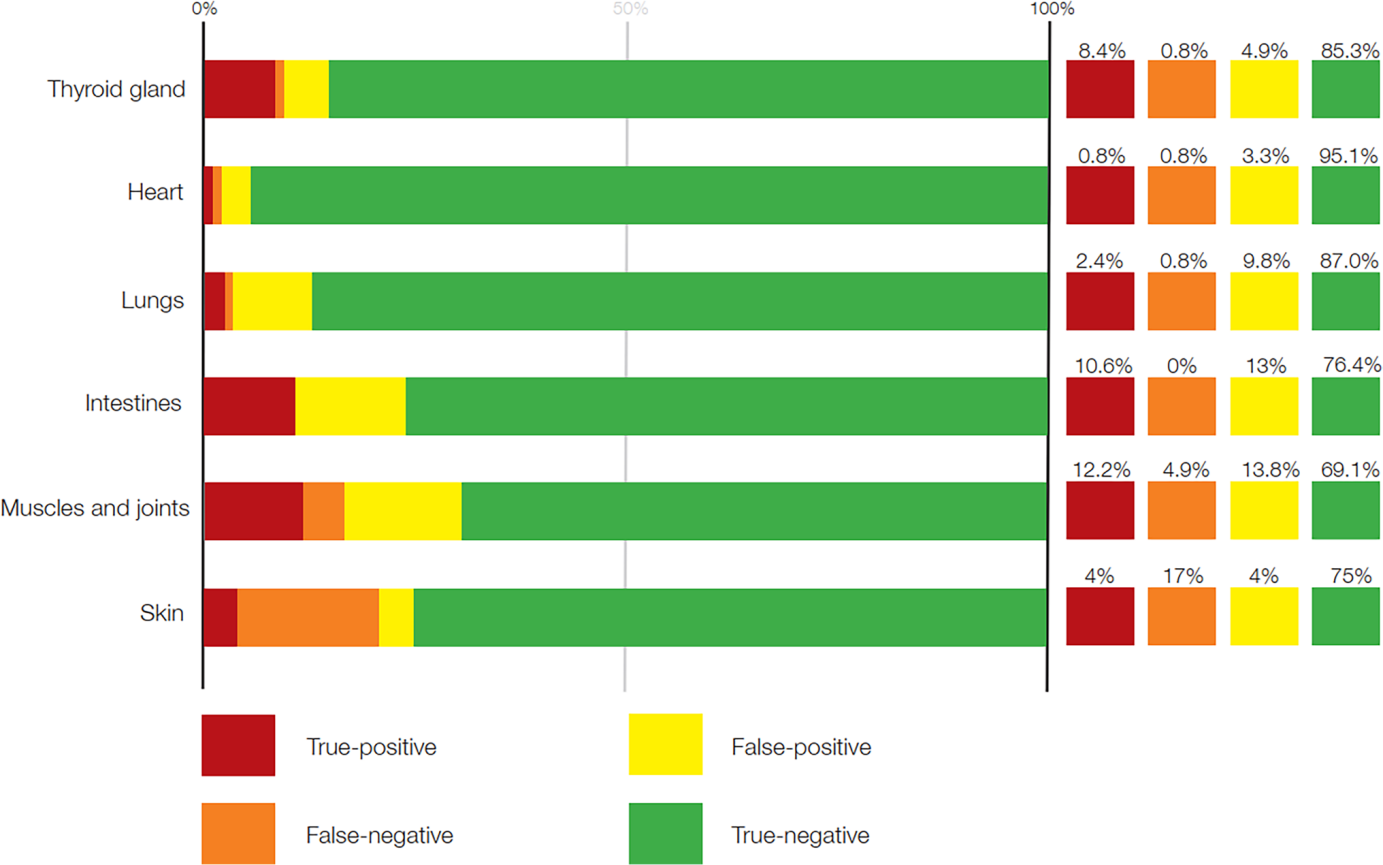
Organ-specific accuracy measures for immune-related adverse events observed on [^18^F]FDG-PET/CT within the first year of immunotherapy treatment in patients with melanoma

**Fig. 3:**
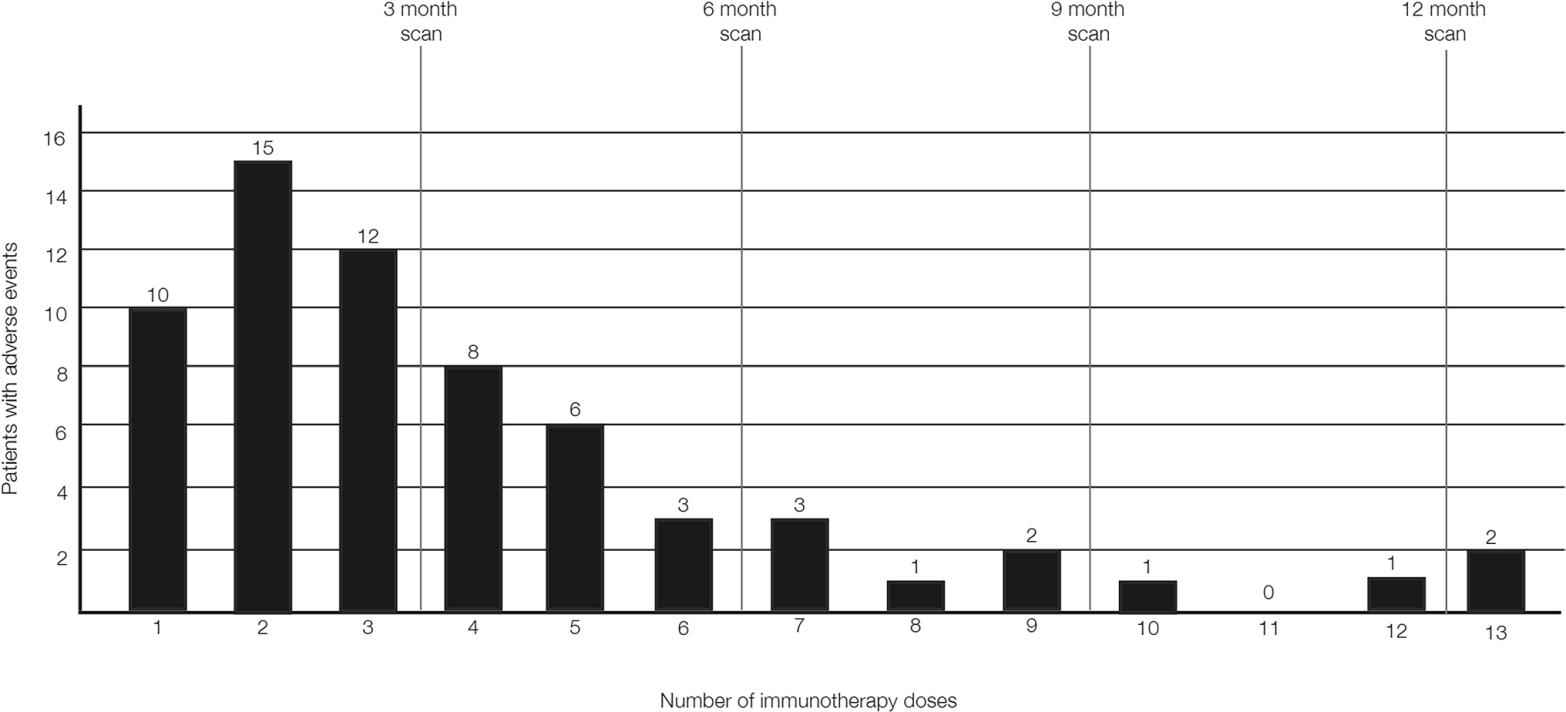
Incidence of immune-related adverse events in patients with melanoma and the number of immunotherapy dose

Treatment-related adverse events occurred in 65/123 patients (52.8%) as judged by the clinicians' reports in the patients' medical records. A total of 86 irAEs were reported in these 65 patients, being most frequent in the skin (26/65; 40%) and in the muscles and joints (21/65; 32.3%). The assessment of organ-specific irAEs by [^18^F]FDG-PET/CT within the first year of immunotherapy is illustrated in Fig. 2. A complete overview of the organ-specific accuracy of [^18^F]FDG-PET/CT in diagnosing irAEs is provided in Table 2. The patients received a median of seven (1–13) doses of immunotherapy. The irAEs were mainly reported within the first three doses in 37/65 patients (56.9%), with the most frequent time-point of occurrence being in relation to the second dose (Fig. 3).

**Table 2:** Accuracy of [^18^F]FDG-PET/CT in diagnosing immune-related adverse events (irAEs) events in specific organs

Involved organs	Positive		Negative		Sensitivity (95% CI)	Specificity (95% CI)	PPV (95% CI)	NPV (95% CI)
	True	False	True	False				
Thyroid gland	11	6	105	1	0.92 (0.62–1)	0.95 (0.89–0.98)	0.65 (0.38–0.86)	0.99 (0.95–1)
Heart	1	4	117	1	0.50 (0.01–0.99)	0.97 (0.92–0.99)	0.2 (0.01–0.72)	0.99 (0.95–1)
Lungs	3	12	107	1	0.75 (0.19–0.99)	0.90 (0.83–0.95)	0.2 (0.04–0.48)	0.99 (0.95–1)
Intestines	13	16	94	0	1.00 (0.75–1)	0.86 (0.78–0.92)	0.45 (0.26–0.64)	1.00 (0.96–1)
Muscles and joints	15	17	85	6	0.71 (0.48–0.89)	0.83 (0.75–0.90)	0.47 (0.29–0.65)	0.93 (0.86–0.98)
Skin	5	5	92	21	0.19 (0.07–0.39)	0.95 (0.88–0.98)	0.5 (0.19–0.81)	0.81 (0.73–0.88)

[^18^F]FDG-PET/CT: Fluorodeoxyglucose positron emission tomography with integrated computed tomography, PPV: Positive predictive value, NPV: Negative predictive value, CI: confidence interval

Of the patients who developed irAEs, 62 required additional treatment, and most patients (36/62; 58.1%) received systemic prednisolone. Among the patients who developed irAEs, 40/65 patients (61.5%) experienced consequences to such an extent and severity resulting in the discontinuation of immunotherapy. In analyses conducted to investigate the correlation between organ-specific irAEs and the discontinuation of therapy, we found that in 10/13 patients (76.9%) with irAEs in the intestines, immunotherapy was terminated. The skin was the organ with the most frequently reported irAEs; 23/26 patients (88.5%) received treatment, and 11/26 (42.3%) discontinued immunotherapy. The patients who suffered from thyroid gland complications were treated with thyroid hormone replacement therapy. Within the included population, 7/123 (5.7%) were diagnosed with diabetes mellitus, and all seven were treated with metformin. Table 3 provides an overview of the patients who either received treatment or discontinued immunotherapy due to irAEs, as well as those who did not require therapy termination.

**Table 3:** Clinical management of patients after the occurrence of immune-related adverse events*

Treatment organs	Patients with irAEs	Treatment received	Immunotherapy	
			Continued	Discontinued
Thyroid gland	12	12 (100)	7 (58.3)	5 (41.7)
Heart	2	2 (100)	0	2 (100)
Lungs	4	4 (100)	1 (25)	3 (75)
Intestines	13	11 (84.6)	3 (23.1)	10 (76.9)
Muscles and joints	21	19 (90.4)	12 (57.1)	9 (42.9)
Skin	26	23 (88.5)	15 (57.7)	11 (42.3)

*Data is shown as frequency (%)

irAEs: immune-related adverse events

Increased FDG uptake on [^18^F]FDG-PET/CT in either the mediastinal or hilar lymph nodes were detected in 21/123 patients (17.1%). Other irAEs that were not included in the tables and/or figures were pancreatitis 1/65 (1.5%), hepatitis 4/65 (6.2%), nephritis 1/65 (1.5%), neuritis 3/65 (4.6%), and sarcoidosis 1/65 (1.5%).

### Accuracy

**Fig. 4:**
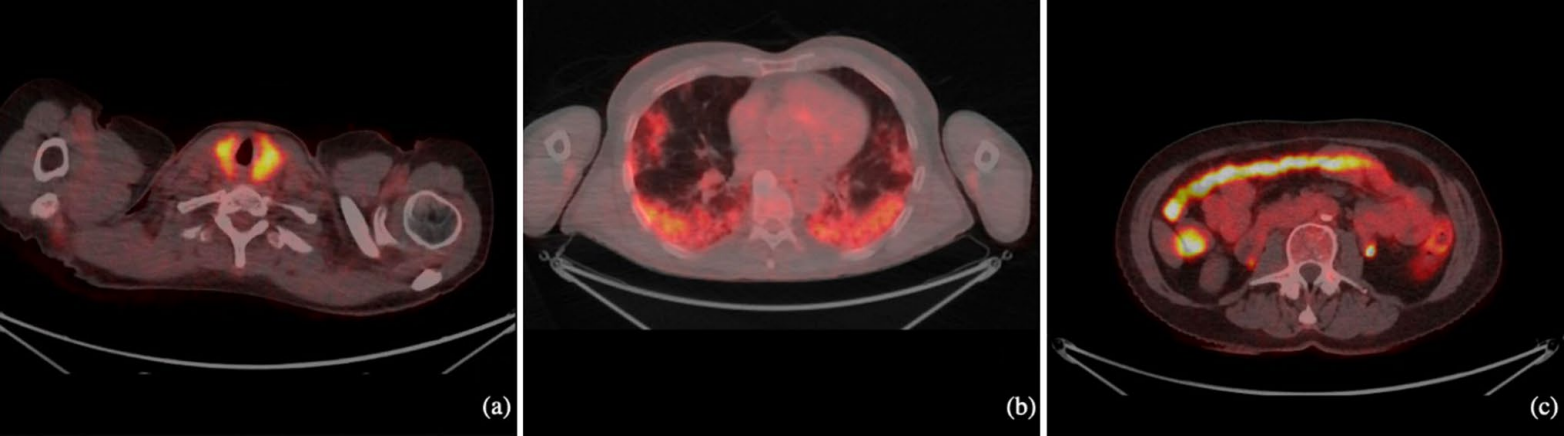
Transaxial [^18^F]FDG-PET/CT scans of three patients diagnosed with true-positive immune-related adverse events after receiving immunotherapy. **a** Thyroiditis, **b** Pneumonitis, **c** Colitis

The highest sensitivity was observed when identifying intestinal and thyroidal adverse events, with values of 100% (95% CI: 75–100%) and 92% (95% CI: 62–99%), respectively. In contrast, the lowest sensitivity was detected in the skin (19%; 95% CI: 7–39%). Generally, the specificities were great, with a specificity of 95% for the skin (95% CI: 88–98%) and thyroid gland (95% CI: 89–98%). Additional results on sensitivities, specificities, PPVs and NPVs are shown in Table 2. Transaxial [^18^F]FDG-PET/CT scans of three patients diagnosed with true-positive irAEs after receiving immunotherapy are presented in Fig. 4.

## Discussion

In this retrospective study on diagnostic accuracy, [^18^F]FDG-PET/CT provided information that could be used to identify irAEs. Of the 123 enrolled patients, 65 experienced irAEs that either required additional treatment or caused discontinuation of immunotherapy. The incidence of organ-specific irAEs, presented in decreasing order, were: skin 26/65 (40%), muscle and joints 21/65 (32.3%), intestines 13/65 (20%), thyroid gland 12/65 (18.5%), lungs 4/65 (6.2%), and heart 2/65 (3.1%). [^18^F]FDG-PET/CT detected 48 true-positive cases, with the highest sensitivity in the intestines and the thyroid gland, where the values were 100% (95% CI: 75–100%) and 92% (95% CI: 62–99%), respectively. In contrast, [^18^F]FDG-PET/CT showed a low sensitivity of 19% in detecting adverse events in the skin (95% CI: 7–39%). In terms of specificity, high values were found in the skin 95% (95% CI: 88–98%) and thyroid gland 95% (95% CI: 89–98%). Muscles and joints had the lowest specificity in this study, with a moderate 83% (95% CI: 75–90%).

In more than half of the patients (56.9%), the irAEs developed before receiving the fourth dose of immunotherapy. According to the Danish guidelines for the follow-up of patients with high-risk melanoma, the first follow-up scan is scheduled to be performed approximately three months after the patient has received the initial dose of immunotherapy [20]. As a result, when the first follow-up scan is performed, the irAE may have been treated, leading to undetectable FDG uptake on [^18^F]FDG-PET/CT. This is also supported by our results showing that out of the 40 patients who discontinued treatment, immunotherapy was terminated in 18 patients (45%) after receiving a maximum of three doses. Detection of irAEs at an earlier stage could potentially guide clinicians to adjust the treatment earlier, which may result in an increased number of patients completing the desired treatment line.

[^18^F]FDG-PET/CT is known for its high sensitivity in detecting metastases; however, inflammation from any cause will also represent increased FDG uptake. Consequently, irAEs generated by PD-1 inhibitors may also be detected [32]. Comparing the scans to their respective baseline scans can aid in detecting subtle changes in uptake or density [31]. We recognise that diagnosing colitis on [^18^F]FDG-PET/CT can be difficult, as normal metabolic activity in the colon might make it challenging to distinguish physiological uptake from inflammation. In asymptomatic patients, increased colonic FDG uptake due to benign causes is common [33, 34]. We found a high sensitivity in detecting irAEs in the intestines (100%); however, the specificity was moderate (85%). These results are supported by a study by Gelston et al., which claims that FDG PET/CT is a valuable imaging method for detecting an increase in gastrointestinal inflammation [35]. According to multiple studies, patients using the oral hypoglycaemic medication metformin have diffusely enhanced tracer absorption in the colon, which may lead to a higher incidence of false-positive cases [31, 36–38]. In this study, 4/16 patients (25%) with false-positive results in the intestines had diabetes mellitus and were undergoing treatment with metformin. Consequently, metformin should be discontinued when the glucose analogue FDG is administered to obtain higher specificity in PET imaging [25].

Thyroid dysfunction typically results in diffuse, homogenous, enhanced tracer absorption on [^18^F]FDG-PET/CT [39], which was also observed in all patients in this study. Thyroiditis can be challenging to diagnose because of its nonspecific symptoms, such as fatigue and headache, and thyroid function tests are recommended for confirmation. Thyroiditis was reported and confirmed by laboratory tests in 12/65 patients (18.5%). The median time of onset after the initiation of immunotherapy was eight weeks (two doses), supported by the findings of another study [40]. In the present study, [^18^F]FDG-PET/CT was highly accurate in detecting irAEs in the thyroid gland, with sensitivity and specificity of 92% and 95%, respectively. [^18^F]FDG-PET/CT has already shown perfect performance in detecting permanent thyroid dysfunction in patients with melanoma treated with PD-1 inhibitors [41], corroborating the findings of our study. Thyroid hormone replacement therapy was required in all 12 patients, and 5/12 patients (41.7%) discontinued immunotherapy after developing thyroiditis, which was due to early-onset toxicity or mixture of other complications.

The [^18^F]FDG-PET/CT scans showed an overall moderate sensitivity in the organs discussed in this study, ranging from 50 to 75%. [^18^F]FDG-PET/CT had poor sensitivity in detecting irAEs in the skin, which was reflected in its low sensitivity (19%) and PPV (50%). In patients with melanoma, such poor results in the skin could potentially be due to a misinterpretation of its findings as disease recurrence, pseudoprogression, or wounds after surgery. Musculoskeletal processes may lead to focal uptake on [^18^F]FDG-PET/CT [42]. The incidence of arthralgia was approximately 9–13% in a study by Benefareni et al. [43, 44], whereas in this study, musculoskeletal irAEs were considered one category and registered in 21/123 (17.1%) patients. Pneumonitis can cause clinical symptoms such as dyspnea or cough, but it can also be asymptomatic [45]. This could be considered for 12 patients who were classified as false-positive when the lungs were investigated. Due to physiologic FDG uptake in the heart, such can occasionally be misinterpreted as myocarditis, potentially resulting in increased false-positive cases.

A systematic review and meta-analysis evaluated the value of [^18^F]FDG-PET/CT for predicting or monitoring immunotherapy response in patients with melanoma [46]. They showed that some of the parameters present promising predictors of the final response to immunotherapy. These results supplement our findings of the accuracy of [^18^F]FDG-PET/CT in the diagnosis of irAEs due to immunotherapy.

In this study we found that 65/123 patients (52.8%) developed treatment-related adverse events. Compared with a similar study [3], adverse events from any cause were reported in 96.9% of the patients, of which 14.4% were considered grade 3 and 4. In our study, the results showed that out of the 123 patients who received immunotherapy, 40 patients (32.5%) discontinued immunotherapy because of irAEs. In contrast, in a study by Weber et al.[3], approximately 10% of patients terminated treatment due to treatment toxicity.

Sarcoidosis-like reactions have been linked to the administration of PD-1 inhibitors and are drug-induced [47]. A drug-induced sarcoid reaction (DISR) is described as a systemic granulomatous tissue reaction that is indistinguishable from sarcoidosis and occurs in close proximity to the initiation of an offending drug [48]. DISR is the most relevant irAE that could be misinterpreted as malignancy because it mimics newly developed hilar and mediastinal lymph node manifestations [10]. Generally, DISR is asymptomatic, and no treatment is required [47, 48]. DISR was identified as mediastinal/hilar lymphadenopathy and was most frequently detected on the first scan after the initiation of immunotherapy [47], comparable to our study, in which 16/21 patients (76.2%) were identified with lymphadenopathy in the first follow-up scan. Our study observed such scans in 21/123 patients (17.1%), and 13/21 patients (61.9%) had no clinically registered irAEs. FDG uptake was increased in both hilar and mediastinal lymph nodes in 10/21 individuals (47.6%). Only 1/21 patients (4.8%) were clinically diagnosed with sarcoidosis.

In the interpretation of [^18^F]FDG-PET/CT scans, it is imperative to differentiate between irAEs, disease recurrence, and other non-specific inflammatory conditions. This distinction is crucial for guiding clinical management decisions and ensuring optimal patient care [49]. While we primarily focused on identifying and characterizing irAEs in this study, it is important to acknowledge the broader clinical context. Recurrence of the underlying malignancy and non-specific inflammation due to conditions like osteoarthritis and nonspecific reactive lymphadenopathy can often present with increased [^18^F]FDG uptake patterns that may overlap with irAEs [31, 50, 51]. Experienced nuclear medicine specialists read the scan in this study to diminish some of these confounding factors.

This is, to the best of our knowledge, the first study to assess the accuracy of [^18^F]FDG-PET/CT in detecting irAEs in patients with melanoma treated with adjuvant immunotherapy. The patients in this study had characteristics reflecting a standard clinical sample of patients from our population of interest, representing daily clinical practice leading to a higher external validity. All patients who met our inclusion criteria were included, which decreased the chances of selection bias.

Several limitations need to be acknowledged in our study. Firstly, the utilization of clinically registered irAEs as our reference standard introduces the possibility of falsely low sensitivity. IrAEs could have been treated or resolved prior to the subsequent [^18^F]FDG-PET/CT scan, thus rendering them undetectable by this modality. Furthermore, given that certain adverse events are asymptomatic, there exists a potential for clinical under-recognition, leading to misleadingly low specificity. It is essential to note that distinguishing between the physiological distribution of FDG in organs such as the intestines, heart, and muscles, and inflammation, can be inherently challenging. The variability in physiological FDG uptake, including factors like medication intake (such as metformin), intestinal motility, and muscle activity, introduces complexities that were not explicitly controlled for in our retrospective methodology. This limitation may have impacted the precision of our interpretations.

Additionally, while our study's design involved experienced examiners, the absence of formal assessment of inter-observer agreement among the three clinicians is another limitation. Despite their proficiency, the potential for interpretation discrepancies exists, which could have influenced diagnostic accuracy. Notably, a limitation intrinsic to FDG PET/CT is its sensitivity in detecting skin inflammation, constrained by resolution limitations. The suboptimal spatial resolution of PET scans can impact our ability to precisely identify and quantify skin-related irAEs.

Furthermore, the assessment of hepatic inflammation related to irAEs was inadvertently omitted from this study. This omission is partly attributed to the challenge posed by the high physiological FDG distribution in the liver, which can potentially obscure subtle inflammatory changes. We recognize the importance of this aspect and acknowledge its absence in our study.

Moreover, the occurrence of a false-negative result in myocarditis detection should be noted. This was due to physiologic FDG uptake in the myocardium, which is a potential limitation of [^18^F]FDG-PET/CT in myocarditis diagnosis.

Given the retrospective nature of our investigation, detailed patient irAE data acquisition was limited to registered medical records. Additionally, only patients who experienced altered clinical management due to irAEs were considered eligible. More comprehensive assessment and diagnosis of adverse events would require para-clinical test results. Applying the Common Terminology Criteria for Adverse Events grading system could further enhance the study's validity.

In addition to our analysis of true-positive cases, it is essential to consider the significance of false-positive and false-negative findings in [^18^F]FDG-PET/CT detection of irAEs. While false-positives may lead to unnecessary concerns and interventions, false-negatives could result in missed opportunities for early intervention [31, 52]. It is noteworthy that the interpretation of [^18^F]FDG-PET/CT scans encounters challenges in distinguishing physiological from pathological FDG uptakes. A substantial limitation in this context is the absence of a definitive reference standard for making this differentiation.

In future studies, it would be clinically beneficial to see whether changing the timeline of the [^18^F]FDG-PET/CT scans would yield different results. Since more than half of the patients develop irAEs after the initial three immunotherapy doses, adjusting the intervals between the [^18^F]FDG-PET/CT scans should be considered. If the scans were performed more frequently initially, it is likely that more irAEs would be detected on [^18^F]FDG-PET/CT, potentially leading to earlier treatment when needed. Additionally, considering that irAE appearance has been linked to the efficacy of immunotherapy, investigating the predictive role of these events in immunotherapy response could potentially reduce patient discontinuation of immunotherapy [53]. This emphasis on early irAE treatment aligns with the need to differentiate irAEs from other conditions. To further enhance the clinical utility of [^18^F]FDG-PET/CT in this context, future research should investigate specific differentiation strategies and diagnostic algorithms for distinguishing between irAEs, recurrence, and other inflammatory conditions. This endeavour will play a significant role in refining the utility of [^18^F]FDG-PET/CT in guiding treatment decisions and monitoring therapeutic responses in oncology patients.

## Conclusion

Our findings indicate that over half (52.8%) of patients who received adjuvant immunotherapy experienced inflammatory adverse events following surgery for high-risk melanoma. This underscores the need for a method for early detection to identify irAEs in order to enhance clinical management. It seems that most patients encountered irAEs before their fourth dose, which may have resulted in either spontaneous resolution or necessary treatment. As a consequence, they could have had undetectable FDG uptake on [^18^F]FDG-PET/CT. In diagnosing irAEs, the [^18^F]FDG-PET/CT proved to be highly sensitive for diagnosing thyroiditis and colitis and highly specific for thyroiditis. Given the clinically acceptable accuracy of [^18^F]FDG-PET/CT in detecting irAEs, it could be valuable to include these evaluations in the scan reports for disease recurrence detection. Confirming the potential benefit of [^18^F]FDG-PET/CT for better clinical decision-making in managing irAEs in melanoma patients requires prospective multicenter studies.
